# Complete financial disclosure for improved transparency in nutrition communication

**DOI:** 10.3389/fpubh.2025.1707013

**Published:** 2025-12-03

**Authors:** Andrew N. Reynolds, Sabita S. Soedamah-Muthu

**Affiliations:** 1 Department of Medicine, Edgar Diabetes and Obesity Research Centre (EDOR), University of Otago, Dunedin, New Zealand; 2 Department of Medical and Clinical Psychology, Center of Research on Psychological Disorders and Somatic Diseases (CoRPS), Tilburg University, Tilburg, Netherlands; 3 Institute for Food, Nutrition and Health, University of Reading, Reading, United Kingdom

**Keywords:** conflict of interest (COI), nutrition communication, financial disclosure, transparency and disclosure, financial support

## 1 Introduction

Globally, the foods we eat and their nutrients are the leading causes of morbidity and mortality (1), making nutrition communication an essential tool for improving health outcomes and lowering healthcare costs. However, not all nutrition communication is delivered based solely on health. Given the ability of nutrition communication to influence self-perception and behavior (2), the accurate disclosure of financial support is fundamentally important to identify an individual's or research group's positionality and vested interest. This is seen as particularly key in nutrition science, where competing interests are high (3–5) and consumer confidence in nutrition science is low (6).

Financial support is important to disclose to understand the risk of bias in nutrition communication, such as that caused by commercial determinants of health. The World Health Organization defines the commercial determinants of health as direct and indirect strategies or approaches used by the private sector to promote products and choices that positively or negatively affect health (7). Nutrition science and its communication are areas where health and commercial interests interact (8, 9). Given the importance of this topic, several previous publications have discussed aspects of increasing scientific rigor and appropriate disclosure of competing interests (10, 11), often with mixed or conflicting views (12–14). Guidelines for recognizing competing interests to maintain scientific integrity and credibility have been proposed with general phrasing such as “parties shall require, in publications and conference presentations, full signed disclosure of all financial interests” (10). However, it has been acknowledged (15) that specific structural guidance on how to disclose competing interests in nutrition communication is lacking (16).

While virtually all scientific journals adhere to their guidelines for disclosing financial support, our experience has shown that such guidelines do not distinguish between disclosure (potential for bias) and acknowledgment. Furthermore, nutrition conferences provide less guidance and do not typically uphold the requirement to list disclosures for all speakers. Here, we have defined useful terminology, identified different types of topic-related financial support, distinguished between what should be disclosed and what should be acknowledged, and proposed a guide to do so. We do this to increase transparency and consistency among researchers, as well as to aid nutrition communicators in revealing funding assistance in an open and transparent manner.

## 2 Terminology and key concepts when disclosing financial support

Scientific integrity and trustworthiness are key to guaranteeing credibility in nutrition science and ensuring accurate health promotion in oral presentations and written work (nutrition communication). Scientific integrity is defined as the commitment to uphold ethical principles, professional standards, and honest practices in the conduct, management, use of results, and communication of science and science activities (17). Trustworthiness is defined as the perceived reliability, trust, or credibility of an individual by others (18). Transparent disclosure of financial support that could influence nutrition communication is essential to maintaining both scientific integrity and trustworthiness.

Throughout this writing, specific terms will be used to mean specific things. Differences in terminology and the use of non-specific terms can be misleading, making shared terminology when disclosing financial support important. Specific terms to use are defined in Table 1, which we adopted or adapted from the current literature. Our use of terms starts with the understanding that the nutrition communicator discloses their financial support, and it is the audience who determines any competing interests. It cannot be the role of the nutrition communicator to state their competing interests, as this is a judgment statement in which they might be biased. The focus of this writing is to disclose financial support in any way relevant to nutrition communication. Financial support can be divided into income or financial support managed through a host institution (university). In-kind support is included within financial support because if the goods/services were not directly provided, then they would have been purchased. Relevant patents (awarded or pending) are also included when disclosing financial support, even if they represent only potential future gain. Relevant financial disclosures should be listed for both the nutrition communicator and the research team behind the work.

**Table 1:** Definitions and preferred word use regarding disclosing financial support.

**Key term**	Definition^*^	**Similar words**
Disclosure	The act of making a fact or information known. This is what is done by the nutrition communicator to their audience.	Declaration of interests.
Competing interests	When one interest can compromise judgment/actions related to another interest.	Conflict of interests, opposing interests, conflicting demands, diverging interests.
Financial support	Monetary or economic benefit, right, or stake that an individual or entity holds, often in relation to a specific asset, venture, or activity.	Financial interests, research support, and income support.
Income	Money (or goods) received personally in exchange for work or activities undertaken or committed to. Can be paid on a regular basis or be a one-off or intermittent payment.	Funding, financial support, salary, consulting fees, and consultancy.
Unrestricted project grant	Project funds where the funder does not have a role in the conceptualization, design, data collection, analysis, decision to publish, or preparation of the manuscript.	Research grant, research funds, project funding, and grant in aid.
Restricted project grant	Project funds are where the funder has any role in any of the conceptualization, design, data collection, analysis, decision to publish, or preparation of the manuscript.	Research grant, research funds, project funding, grant in aid.
In-kind support	Non-monetary contributions, such as goods or services, are provided instead of money. These goods or services would otherwise need to be paid for.	Material aid, in-kind donation, physical resources.

^*^Definitions were adapted or adopted from the existing literature by the authors.

Finally, disclosure will differ between restricted project funding and unrestricted project funding. Project funding is only unrestricted when the funder has no role in the study's conceptualization, design, data collection, analysis, the decision to publish, or communication of results, such as presentations or publications. These categories, which a funder may influence or bias nutrition science, are those indicated in the Nature Portfolio's Competing Interests Policy (19). In contrast, unrestricted project funding provides the freedom to conduct and present research without influence from that funder.

## 3 Types of topic-related financial support to disclose in nutrition communication

It is important to acknowledge that there are various forms of competing interests that could influence nutrition science and communication (20), with the scope of this article focused on disclosing an individual's or research team's financial support. Non-financial competing interests may be equally important (21, 22), but these should be reported separately and distinctly from financial ones. Mixing financial and non-financial competing interests will likely dilute the visibility of financial disclosures. Furthermore, listing funding and positions not related to the topic of nutrition communication or where no competing interest can exist will also (intentionally or not) dilute the visibility of financial disclosures. Listing credible funding sources from unrelated projects is another way the visibility of relevant financial disclosures may (intentionally or not) be diluted.

Understanding the relevant types of financial support is crucial when disclosing this information. Financial disclosures should relate to both income and money or goods/services passing through an institution (university) relating to the topic. Topic relevance is broader than project relevance when disclosing and removes much of the need to specify a timeframe within which disclosures should be stated. Instead, any topic-relevant funding received in the past should be disclosed.

### 3.1 What to disclose first?

Income is money (or goods) received personally in exchange for work or activities undertaken or committed to. Income includes both base salary and additional sources paid to an individual and should be the first disclosure stated when relevant to the topic of communication. All sources of income/salary relevant to the topic should be disclosed, e.g., salary, royalties, honorarium, directorship, trust, and/or dividend payouts. This includes anticipated or committed future income not yet paid. If an individual receives multiple sources of relevant income, it is useful to order them by amount (without the need to state amounts). It is also useful to indicate the source of the income [e.g., university, government, other (topic-unrelated), or other (topic-related)] when disclosing. Nutrition communicators should focus on topic relevance and disclose all sources rather than reporting only if above an arbitrary amount, as has previously been considered (23).

### 3.2 What to disclose next?

After income, relevant financial support passed through or managed by an institution, such as a university, should be disclosed next. Such financial support includes project grants and conference attendance support and can include honoraria, speakers' fees, director, or board fees that are not paid as income.

The first of these to disclose is topic-relevant restricted project funding, where the funder was involved in any aspect of study conceptualization, design, data collection, analysis, the decision to publish, or communication of results. Relevant restricted project funds must always be disclosed, as this seems to be a common pathway to introducing bias into analyses and interpretation (4, 5, 24). Like income, the source of restricted project funds should also be categorized [university, government, other (topic-unrelated), or other (topic-related)]. Following income and restricted project funding, certain other topic-relevant financial support passed through or managed by an institution can then be listed (e.g., conference attendance support, honorarium/director fees/board fees/speakers' fees, shares/trusts/dividends/royalties, and in-kind support), followed by patents. Finally, if the nutrition communicator has family members receiving topic-related financial support, including nutrition science-related trusts/foundations or employment positions, these should be disclosed.

This writing assumes all financial relationships are disclosable. However, it is possible that some nutrition communicators may have non-disclosure agreements or confidentiality agreements in place. In these situations where certain disclosures cannot be itemized, and in line with the guidance of the Nature Publishing Group, authors should state: “The authors declare that they are bound by confidentiality agreements that prevent them from disclosing their competing interests in this work.” There is no possible guidance where nutrition communications are prevented by confidentiality agreements from disclosing both competing interests and the presence of a confidentiality agreement; however, such agreements can be in place.

### 3.3 What financial support should not be disclosed, but acknowledged?

When excess or unnecessary information is disclosed, it can dilute or obscure the disclosure of important information for the audience. For this reason, it is important for nutrition communicators to understand what must be disclosed and what might instead be acknowledged. Principally, unrestricted project funds, irrespective of source, should not be included in disclosures. Given that truly unrestricted project funding provides the freedom to conduct and present research without any influence from that funder, there is no competing interest to ascertain. Similarly, it is recommended not to list disclosures for financial support passed through or managed by an institution, such as a university, that comes from university, government, or other (topic-unrelated) funders when competing interests cannot exist. Instead of disclosing, these types of financial support could be acknowledged on a separate slide or publication section alongside the relevant grant number or identifier.

**Figure 1:**
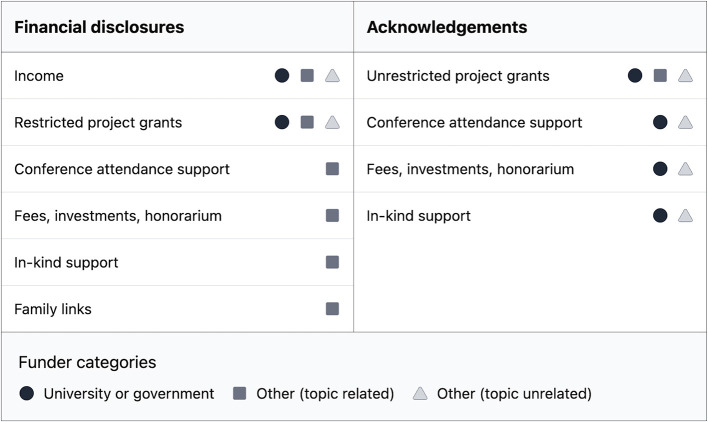
Proposed way forward for disclosing or acknowledging topic-related financial support in nutrition communication. *While governments might place greater value on commercial benefits than health benefits, and that funding in this situation could introduce a competing interest for the nutrition communicator, hopefully is an exception left to the individual's discretion to report.

A guide to disclosure and acknowledgment of financial support is shown in Figure 1. A summary of this guidance is: disclose relevant income first, ordered by amount, and state the source. State relevant restricted project funding second, including funding sources. State other relevant funding sources, third, including the source. State family disclosures, if relevant. If required or relevant, state non-financial disclosures separately from financial disclosures. Do not mention in disclosures unrestricted project grants or funding sources where competing interests cannot exist, or funding support not relevant to the topic, but mention these in the acknowledgments.

## 4 Further reading on competing interests

For those interested in scientific rigor, this online list of relevant articles is useful (25), as are more recently published resources (22).

## 5 What about non-financial disclosures?

As previously stated (21, 22), concepts such as social/cultural/strong lifestyle preferences might also introduce competing interests in nutrition communication. While not a focus of this article, individuals should seek advice from relevant conferences or journals on whether non-financial interests should be disclosed after and separate from financial support.

## 6 Conclusion

Nutrition science and communication are areas where health and commercial interests interact, with a clear potential for detrimental impacts on human health. The appropriate disclosure of financial support in nutrition communication is one area where specific structural guidance is lacking. We have provided terminology and a structure to support systematic, clear reporting on financial support. The purpose of this study is to add transparency in disclosing financial support, increasing scientific integrity and trustworthiness in nutrition communication. However, it remains the responsibility of the nutrition communicator to disclose their funding sources in an accurate, truthful, and open manner.
